# Complete mitochondrial genome of Pallas’s Leaf Warbler (*Phylloscopus proregulus*)

**DOI:** 10.1080/23802359.2017.1403867

**Published:** 2018-02-12

**Authors:** Shu-Yu Jiao, Zheng-Xi Liu, Feng Yu, Ji-Yuan Yao, Yu-Mei Li, Shou-Qing Yan

**Affiliations:** a College of Animal Science, Jilin University, Changchun, PR China;; b Animal Science and Technology College, Jilin Agricultural University, Changchun, PR China

**Keywords:** Pallas’s Leaf Warbler, mitochondrion, genome

## Abstract

In the present study, the complete mitochondrial DNA sequence of Pallas’s Leaf Warbler (*Phylloscopus proregulus*) was determined for the first time. The mitochondrial genome of Pallas’s Leaf Warbler is a circular molecule of 16,880 bp in size and contains 13 protein-coding genes, 2 rRNA genes, 22 tRNA genes and 2 control regions. The base composition is 32.7% for C, 14.3% for G, 30.0% for A and 23.0% for T. These data will be useful for studying the genetic diversity within the species of Pallas’s Leaf Warbler and phylogenetic relationships among different Phylloscopidae species.

Mitochondrial DNA has been playing important roles in population genetic and phylogenetic analyses because of its characteristics of small size, fast substitution rate and maternal inheritance pattern (Wada et al. 2010; Statham et al. 2011). The Pallas’s Leaf Warbler (*Phylloscopus proregulus*) is one small insectivorous species belonging to the genus *Phylloscopus* of family Phylloscopidae (Martens et al. 2004; Lei et al. 2010). Up to date, only partial mitochondrial sequence for Pallas’s Leaf Warbler is available. In the present study, we amplified and sequenced the complete mitochondrial DNA of the Pallas’s Leaf Warbler for the first time.

An adult Pallas’s Leaf Warbler was collected from Jilin City of Jilin Province in China and stored in Zoological Specimen Museum of Jilin Agricultural University. Genomic DNA was extracted from blood sample using the TIANamp Genomic DNA Kit (TIANGEN, Beijing, China) according to the manufacturer’s protocol. The complete mitochondrial genome was amplified by LA-PCR with six pairs of primers designed according to the known DNA sequence of Pallas’s Leaf Warbler in GenBank. The total mitochondrial genome of Pallas’s Leaf Warbler is a circular DNA molecule with a length of 16,880 bp and the base composition is 30.0% for A, 32.7% for C, 14.3% for G and 23.0% for T. The sequence has been deposited in GenBank (Accession no. MG189603). The mitochodrial genome of Pallas’s Leaf Warbler contains 13 protein-coding genes (PCGs), 2 rRNA genes, 22 tRNA genes and 2 control regions (D-loop regions). Except for ND6 and 8 tRNA genes, other genes are coded on the H strand.

The DNA data of 13 PCGs of *Phylloscopus proregulus* and other 10 Passeriformes species were used for constructing the phylogenetic tree. A neighbour-joining (NJ) tree was built using MEGA 5 (MEGA Inc., Englewood, NJ) with 1000 bootstrap replicates. As indicated by the trees (Figure 1), *Phylloscopus proregulus* has closer affinity to *Phylloscopus inornatus* than to other species analyzed in this study. The result is similar to the previous study (Tietze et al. 2015). The sequence data will be useful for studying the genetic diversity within the species of Pallas’s Leaf Warbler and phylogenetic relationships among different Phylloscopidae species.

**Figure 1.**
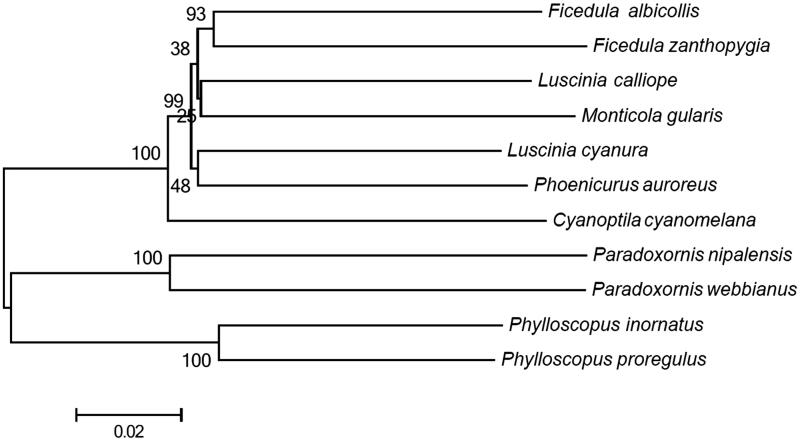
A neighbour-joining (NJ) tree of 11 species was constructed based on the data set of 13 concatenated mitochondrial PCGs using MEGA 5 with 1000 bootstrap replicates. Sequence data used in the study are the following: *Phylloscopus inornatus* (KF742677), *Paradoxornis nipalensis* (NC_028437), *Luscinia cyanura* (KF997864), *Monticola gularis* (NC_033536), *Paradoxornis webbianus* (NC_024539), *Ficedula zanthopygia* (JN018411), *Cyanoptila cyanomelana* (HQ896033), *Luscinia calliope* (HQ690246), *Phoenicurus auroreus* (NC_026066) and *Ficedula albicollis* (NC_021621).
